# Supplementary material for: Pathophysiological Significance of Neutrophilic Transfer RNA-Derived Small RNAs in Asymptomatic Moyamoya Disease
Source: Cells. 2021 May 1;10(5):1086. doi: 10.3390/cells10051086 (PMC8147334; doi:10.3390/cells10051086)

2D Structure	Local AU	Position

```
326                                  341
                    8mer-1a
5 ' - c a t c a A C A A C C A G G G a - 3 ' utr
                |   |   | | | | | | |
                |   |   | | | | | | |
3 ' - u g a u c U G G U G G U C C C u - 5 ' tRF
      16 15 14 13       7 6 5 4 3 2
       3' Pairing          Seed
```

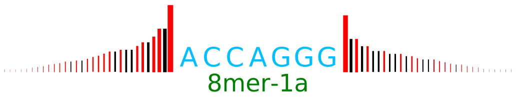

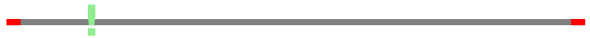

Supplement: Supplementary file 1 [file cells-10-01086-s001.zip › cells-1149161 3rd supplementary materials/Data Supplements/Data Supplement V.zip!/Data supplement 6-mRNAs targets of tsRNAs/tsRNA-Glu-CTC-003/Diagrams/tRF-Glu-CTC-003_vs_ENST00000534668(MS4A1).pdf]
